# Grand Theft Auto-Based Cycling Simulator for Cognitive Enhancement Technologies in Dangerous Traffic Situations

**DOI:** 10.3390/s23073672

**Published:** 2023-03-31

**Authors:** Julius Schöning, Jan Kettler, Milena I. Jäger, Artur Gunia

**Affiliations:** 1 Faculty of Engineering and Computer Science, Osnabrück University of Applied Sciences, DE-49076 Osnabrück, Germany; 2 Faculty of Philosophy, Jagiellonian University, PL-31-007 Krakow, Poland

**Keywords:** cycling simulator, accident prevention, cognitive enhancement technologies, realistic traffic simulation, game engine

## Abstract

While developing traffic-based cognitive enhancement technology (CET), such as bike accident prevention systems, it can be challenging to test and evaluate them properly. After all, the real-world scenario could endanger the subjects’ health and safety. Therefore, a simulator is needed, preferably one that is realistic yet low cost. This paper introduces a way to use the video game *Grand Theft Auto V* (GTA V) and its sophisticated traffic system as a base to create such a simulator, allowing for the safe and realistic testing of dangerous traffic situations involving cyclists, cars, and trucks. The open world of GTA V, which can be explored on foot and via various vehicles, serves as an immersive stand-in for the real world. Custom modification scripts of the game give the researchers control over the experiment scenario and the output data to be evaluated. An off-the-shelf bicycle equipped with three sensors serves as a realistic input device for the subject’s movement direction and speed. The simulator was used to test two early-stage CET concepts enabling cyclists to sense dangerous traffic situations, such as trucks approaching from behind the cyclist. Thus, this paper also presents the user evaluation of the cycling simulator and the CET used by the subjects to sense dangerous traffic situations. With the knowledge of the first iteration of the user-centered design (UCD) process, this paper concludes by naming improvements for the cycling simulator and discussing further research directions for CET that enable users to sense dangerous situations better.

## 1. Introduction

Safety is one of the leading values for both the individual and society. The importance of safety and its effects are indicated by axiological considerations and psychological and economic concepts: safety and security are basic needs in the well-known hierarchy of needs proposed by Maslow [1], and numerous surveys also confirm them as a part of social well-being [2]. In our modern, motorized society, it is imperative to ensure the road safety of vehicle drivers, pedestrians, and cyclists. Cyclists are particularly exposed to the danger of other, often larger, road users. For example, according to police statistics in Poland, in 2020, there were 3768 accidents involving cyclists, including 249 deaths and 3403 injuries, and in 2021, there were 3513 accidents, including 185 deaths and 3192 injuries [3]—the situation is similar in other European countries. Many of these accidents were caused by an incorrect distance estimate by the people involved.

In the context of ensuring safety, the issue of determining the distance is difficult to assess. Both vehicle drivers and cyclists struggle to estimate or recognize the safe distance between two road users. European legal regulations state maintaining a distance of at least 1.5 m [4]. A cyclist has difficulty assessing this distance due to a limited field of view and an inability to see what is behind their back.

Advanced information technology could potentially help inform cyclists about the current road situation, helping them to assess the danger. However, users must test such systems thoroughly before they can be used. Ideally, these user tests would be performed in the real world, but this is inherently dangerous. Therefore, it is necessary to build a system that simulates real traffic objects [5] and situations as closely as possible while keeping the user safe.

This paper outlines the creation of a safe simulator by using the video game *Grand Theft Auto V* (GTA V) as a base. Using this simulator, two early-stage cognitive enhancement technologies (CETs) helping cyclists to identify danger are evaluated. The CETs evaluated are interface concepts that enhance a cyclist’s awareness of dangerous traffic situations. The focus is on making the user aware of the trucks in their proximity that could pose a danger, thus preventing accidents. A user test is applied to assess the effectiveness of this concept.

The objectives presented in the article are (1) showing that wearable technologies are an essential tool for cognitive enhancement concerning human safety, (2) an indication that CETs require multifaceted testing, (3) arguing that for the initial design of CETs for road safety purposes, they can be tested on inexpensive simulators based on computer games, which allows researchers to quickly prove how a given CET system properly affects human cognitive abilities, without putting the subject in dangerous real road situations.

There are multiple reasons to use a video game environment as a simulator. For developing CET in the early iterations, the game GTA V is chosen for the following reasons:

1. GTA V is an off-the-shelf game bringing realistic motorists and cyclists as well as road environments;
2. GTA V is well suited for user experience research because it provides a repeatable environment;
3. GTA V comes with a physics engine that includes realistic weather conditions, trajectories of road users even in crash situations, and sound directions;
4. Using a well-known game, such as GTA V, as a simulator allows the user to express his or her needs better due to the known environment.

We also recognize the limitations of this approach, such as not taking into account specific legal road rules or the advanced interaction between vehicles moving in traffic, pedestrians, and the environment. However, for the pilot testing of the effectiveness of CET, these aspects are not necessary to be taken into account and could even hinder the identification of key aspects of cognitive enhancement for the study.

This paper is organized as follows. In Section 2, the related research is reviewed. Section 3 presents the simulation objectives and a detailed specification of the system. Section 4 presents the concept design based on the user-centered design cycle, followed by its implementation for the cyclist’s safety in the traffic simulator in Section 5. The results and discussion are presented in Section 6, and in Section 7, further extensions of the simulator based on the assumptions of CET are shown, respectively, followed by the conclusions in Section 8. In addition, a technical Appendix A is enclosed.

## 2. Related Work

This section reviews the related works on the usage of technologies that enhance human cognition abilities for increased safety, cf. Section 2.1. In Section 2.2, papers on the usage of computer games as a simulator are reviewed. The computer game as a simulator approach is later used for prototyping the CET systems to ensure safety for cyclists.

### 2.1. Cognitive Enhancement for Increased Safety

Humankind has evolved to be equipped with instinctive reactions to protect it from danger; for example, when perceiving a snake, a bodily reaction triggers fear, prompting the human to flee [6]. However, safety and its counterpart danger are often challenging to assess quantitatively. People are often unaware that situations are unsafe; it also requires knowledge and mental work to assess the situation correctly. Since people are not yet equipped with such an appropriate safety sense, technology can supplement this sense.

The idea of cognitive enhancement includes general assumptions that refer to improving intellectual abilities and perception, including a sense of safety. Sandberg and Bostrom [7] defined cognitive enhancement as the amplification or extension of the core capacities of the mind through the improvement or augmentation of internal or external information processing systems. It includes improved intelligence and attention, strengthening creativity and memory, and expanding the perceptual range. Cognitive enhancement also refers to the potential benefits of increasing cognitive function beyond ordinary or average capacity and improving control, emotion, or motivation [8]. The deployment of advanced technological tools and techniques is a key feature of cognitive enhancement, as well as its voluntary use by healthy people [9].

Different techniques can be used for cognitive enhancement, including biotechnological and information technology (IT) systems [10]. In recent years, more and more IT systems have been developed to enhance people’s cognitive abilities. These systems tend to enhance people’s basic intellectual abilities, such as memory or attention [11,12], and also enhance people’s more subtle abilities, such as the aesthetic and moral senses [13], motivation and decision processes [14], emotional attitude [15], visual attention and decision [16], and the learning process [17]. For these enhancing purposes, technologies such as virtual reality (VR), augmented reality (AR), or mixed reality (MR), as well as intelligent agents, such as robots, and other immersive technologies, are often used.

In recent years, several extended reality (XR) technologies, i.e., AR, VR, and MR, have been developed to enhance visual perception, changing our behaviors, phobias, and habits. For example, AR with deep learning algorithms has been shown to enhance the visual inspection of some objects [18]. AR and visual prompting techniques can also manage attention in autism therapy [19], and AR with computer-aided projected information can increase vision correction and readability under strong ambient light [20]. AR glasses that simulate reduced acuity and contrast can be used in rehabilitation to restore central vision in patients with age-related macular degeneration (AMD) [21], and AR wearables have been applied with positive effects in physical education on motor skills learning [22].

These interactive technologies that influence users’ cognition can also be used in road safety. Systems to ensure better detection coverage of the surroundings can support many vehicles [23,24]. AR can support safe driving at night by aligning information with the driver’s eye [25] or can help the driver recognize signs on the road better [26]. Some AR solutions are dedicated to cyclists that help with speed information, setting the path or warning signs [27], and systems that enhance cyclists’ attention and visual perception [28]. Undoubtedly, in the future, AR/MR systems that take into account the impact on other sensory perceptions beyond just visual perceptions should be considered.

### 2.2. Computer Games as Simulators in Research

Many people still associate video games with entertainment or as something for kids and adolescents. However, in reality, computer games have already found their way into the field of education and research. Like in this work, many studies use computer games as a simulator. Disregarding video games as just silly entertainment is not contemporary. Instead, they should be seen as an opportunity for research and may even influence professional careers. Nevertheless, even on a smaller scale, it is clear that video games play a more prominent role and have a more significant impact on their players than is evident at first glance. Different types of games could enhance the skills of their players, with puzzle platform games that could improve their spatial skills, role-playing games that help with organizational and planning skills, and strategy games that affect the problem-solving and spatial skills of players [29].

Although the real world is, of course, as realistic as it gets, running repeatable and safe experiments is not always possible. Weather conditions and lighting alone can be problematic when running the same experiment on multiple days. Then, there are safety concerns, especially in traffic and accident prevention, where users would have to be put in a dangerous situation for the test to be very realistic, which is not feasible. Add to that the fact that software is iteratively prototyped according to the user-centered design process (UCD) [30], and it becomes clear that subjects would have to be endangered repeatedly, making a realistic real-world test of such products unfeasible.

On the other hand, computer games can be an ideal and safe environment for running simulations in both the technical and social sciences, as scenarios can be highly controlled and repeatable, and the users are kept safe. Other projects have used games as such a simulation environment. In the context of social simulations, games such as *Second Life* or *AltspaceVR* were used to conduct classes in virtual conditions, with custom maps for the classroom [31,32]. There are learning opportunities for educational and social skills in games such as *Minecraft* or *Roblox* [33]. In the case of *Roblox*, young players have the opportunity to simulate social situations, such as conducting parliamentary sessions or celebrating holy mass in a virtual church [34].

Video games, where the experiment modules can be built directly into the game’s environment, can work exceptionally well for simulations. Some examples of these games are the economic game *SimCity*, the life simulator game *The Sims*, or the sandbox game *Minecraft*. *Minecraft*, as a multiplayer sandbox game, can be used especially well for multi-perspective simulations. An example used AI techniques, such as reinforcement learning and planning libraries, for robotics simulation within the world of *Minecraft* [35]. In other research, the game is identified as an optimal solution to represent a culturally built environment, in which a semiautomatic production path was used to transfer semantic LIDAR data to the *Minecraft* environment [36]. So *Minecraft* offers a unique opportunity for students to display their creativity and understanding of concepts in more feasible ways than if they were attempted in the real world [37]. It can also serve as a massive multi-user emulated environment for research [38].

In the context of traffic situation simulations, racing games have a promising effect, as findings suggest that racing games can simulate real-life driving. After subjects played the car game *StuntRally*, researchers attempted to identify the users’ characteristic driving profiles [39]. Fischer et al. [40] found that subjects who played a racing game subsequently reported higher accessibility to cognition and a positive effect associated with risk-taking than subjects who played a neutral game. Furthermore, it was possible to train modules for autonomous cars on the driving patterns of players [41,42,43].

The game used for the simulator shown in this paper is GTA V. GTA V, and its previous versions are action–adventure games played from a third-person or first-person perspective. The player takes the role of a criminal and can walk around like a pedestrian or drive various vehicles, from bicycles to cars, boats, and planes. Despite the controversial scenario of being a criminal, and the 18+ PEGI rating, the game has high-quality graphics and realistic environment physics. Several studies have used GTA in some way. An example is where researchers used GTA for vehicle calculation and to follow the best route from a source to a target node in any place on the map [44]. In other research, the virtual environment of this game was used to train and test convolutional neural networks for safe driving and is expected to provide an efficient and well-defined foundation for training and testing trained networks [45]. Furthermore, driving behaviors in GTA were used to train AI to plan view networks for autonomous driving [46]. The GTA environment was also used for an optimal traffic simulation [47]. Many game modifications (mods) have been written for GTA V. One of such mods is *GT Bike V*, which allows the player to control the game with a smart bike trainer or a cycling power meter [48]. Lastly, although it is not focused primarily on the traffic scenario, it is also worth mentioning that GTA was used as a pedagogical tool for ethical analysis in which moral dilemmas could be simulated [49].

## 3. Requirements Analysis of the Simulator

The requirements analysis and specification cover the first two steps of the UCD cycle. The simulator objectives cf. Section 3.1, user groups cf. Section 3.2, the context of use cf. Section 3.3, and user requirements cf. Section 3.4 are summarized below.

### 3.1. Simulator Objectives

**Figure 1:**
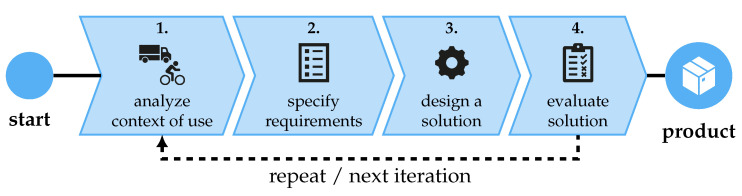
User-centered design (UCD) cycle, cf. [30,50].

The simulator aims to create a realistic bicycle simulation to support testing the concepts of CET in a secure environment. While not endangering the subject, dangerous traffic situations, such as passing trucks in close proximity to the cyclist, will be simulated in a realistic and repeatable way. The simulator will be developed using the UCD cycle. The UCD organizes the development into a cyclic, iterative process, visualized in Figure 1. In the current development stage, the UCD cycle is repeated one and a half times. Therefore, the objective of the simulator is analyzed, specified, developed, evaluated, and new requirements based on the evaluation are identified.

### 3.2. User Groups

**Figure 2:**
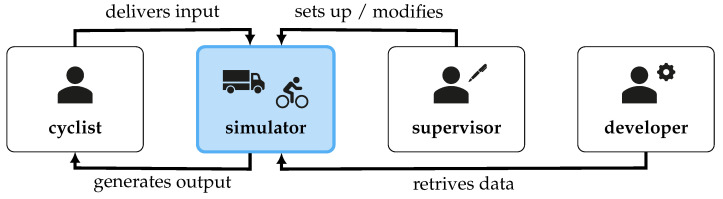
User groups and their interaction with the simulator.

The system developed using the UCD cycle is the bike riding simulator, which serves three main user groups, as illustrated in Figure 2.

**Cyclist:** The cyclists are the main focus group using the simulator. As the name suggests, the user from this group must be able to ride a bicycle before using the system. A cyclist should intuitively know how to interact with the simulator. Therefore, the system should closely emulate normal bicycle controls and must be easy to learn. It should also not pose any real risk to the cyclist. However, it should induce a feeling of being at risk when simulating a dangerous traffic situation. Consequently, it has to immerse the cyclist in being realistic.

**Supervisor:** The supervisor will be present during the simulation. The supervisor has to set up the system and supervise the simulation. They want to give each cyclist—subject—a very similar experience. Therefore, the simulation has to be highly repeatable. During an active simulation, the supervisor also wants to be able to switch between different traffic scenarios. Furthermore, the supervisor works under a time constraint during the simulation, as they must run a user test in a designated time slot, ensuring the smooth handling of the subjects during the evaluation of, for example, CET concepts. Therefore, the system setup and any modifications should be quick and easy.

**Developer:** The developer user group describes the developers of the CET concepts that will be evaluated while using the simulator. The developer is not actively interacting with the simulator during run-time. They interact with it by proxy through whatever CET concept they have designed. For them, the simulator has to offer a way of retrieving data about the currently simulated traffic situation. The data have to be relayed to their concept device in real time. Furthermore, developers want to be free in their choice of the user interface. Therefore, the input collection for the simulator cannot exceed a cyclist’s interactions with a regular bicycle. It must be discrete and have no intrusive sensor assemblies to collect user input.

### 3.3. Simulation Context

Traffic simulations can be notoriously complex, especially in a city [51]. At any given time, there can be various road users in a multitude of vehicles. They all move individually with different velocities, directions, and trajectories that are hard to predict. All road users must also react to each other, adhere to traffic regulations, and avoid collisions if possible. In addition, there is a myriad of environments with different road and weather conditions that influence and define traffic situations. A simulation of such an intricate system is consequently just as complex.

**Figure 3:**
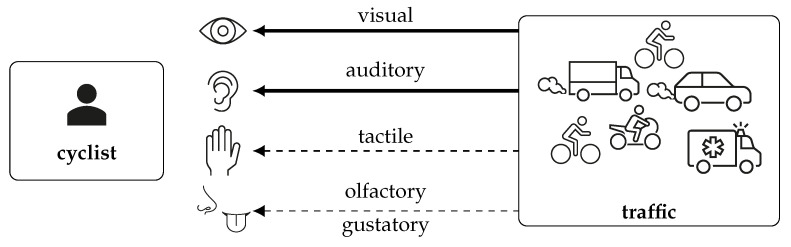
Complexity of a traffic situation and a user’s ability to precept it, cf. [52].

Any traffic situation offers many sensory inputs, especially for cyclists, as they do not have a vehicle exterior around them to protect them. Figure 3 visualizes the sensory input channels and their importance to the cyclists. The visual and auditory senses are the most occupied by traffic, as indicated by the arrows’ strength. Cyclists use their visual sense to recognize vehicles, pedestrians, or other important objects to estimate other road users’ distances and speeds, identify and interpret traffic signs, and analyze the road trajectory in front of them. The auditory sense is used to identify road users that are not yet visible, to estimate the distance to and direction of other vehicles, to register danger in, e.g., the form of car horns, or to determine human speech by either analyzing its content for information and danger clues or by identifying the position and distance to it, to avoid collisions with pedestrians. The tactile sense can help a cyclist assess the state and safety of the road and weather conditions or estimate the distance to surrounding vehicles by interpreting their air gusts. Lastly, cyclists can also use their olfactory and gustatory senses to identify cars through their exhaust fumes or a gasoline smell.

By looking at these examples, it is clear that the visual and auditory senses are the most important for a cyclist and the most important in accident prevention, as they allow cyclists to react and move out of the way before another vehicle is close enough to make contact. The olfactory, gustatory, and tactile channels also get stimulated in traffic but are mainly based on close proximity to a source. The proximity makes them less important for accident prevention. Since accident prevention is the experiment’s focus, a simulator must provide a good visual and auditory representation of traffic to ensure that the simulation is close enough to reality.

### 3.4. User Requirements

The analysis of the different user groups leads to the list of requirements seen in Table 1. For cyclists, the system must be safe, intuitive, and realistic. Supervisors want the simulations to be repeatable and modifiable in a quick manner. Developers want the user input collection to be discrete and non-intrusive. They also require an interface to collect real-time data on the currently simulated traffic situation.

## 4. Concept Design

This section describes the design decision of the third stage in the UCD cycle. Thus, it documents the possible designs and the design decisions.

### 4.1. System Choice

Three possible solutions to simulate traffic situations were identified.

**Prop Simulation:** Practical props, such as a person moving a cardboard device, are used as a truck while the cyclist rides a real bicycle.

**Video Simulation:** Recorded videos of specific traffic situations are used to display the situation on a screen while the cyclist is placed on a stationary bike.

**Computer Simulation:** Computer simulations, such as a video game, are used to bring interactive footage to a screen. The stationary bike is modified so that it can be used as an input device for the program.

Table 2 brings these options in juxtaposition with each other so that, with the attributes visual realism, auditory realism, interactivity, simplicity, and repeatability, a decision matrix is created.

Based on the decision matrix, computer simulations promise to be the most true-to-life experience for cyclists due to suitable sounds and visuals and the ability to interact and move away from dangerous trucks. Therefore, this design decision is followed. However, the time required to implement such a simulator from scratch will be tremendous. By combining available projects, the implementation time can be reduced. One project, found after online research, turns a bicycle into a gamepad controller with low effort [53]. The bicycle gamepad controller will lead to realistic traffic simulation combined with conventional video games, such as GTA and Truck Simulator. If the design decision of the computer simulation cannot be implemented, the video simulation design might be considered as fallback.

### 4.2. Input Device

The cyclist’s input must be captured and transferred into the game to achieve intractability. A simple solution for that is to use a regular gamepad controller. This solution is discarded, as it does not offer a realistic cycling interaction. Instead, the goal is to use a bicycle with which the subject can interact. The inputs will then be transformed into controller signals through various sensors. These sensors have to capture the steering angle of the handlebar, the speed of the tires or paddles, and the braking force. A limited number of similar projects have already tried to achieve that [53,54,55]. Table A1 compares the inputs captured by these projects. Based on this comparison, the input device should be able to capture all inputs with a level from 0% to 100%. The steering will be captured from −100% to 100%.

**Figure A1:**
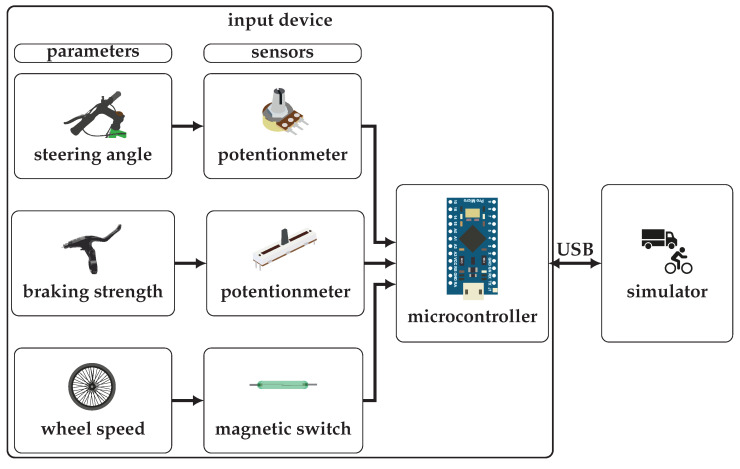
Architecture of the input device.

Figure A1 shows the hardware architecture for the input device. The architecture includes two potentiometers to translate the steering angle of the handlebar as well as the braking strength into electrical signals and a magnetic sensor for capturing the wheel’s speed. All sensors are wired to an Arduino Pro Micro microcontroller board that captures the electrical input signals and translates them into gamepad controller signals for the computer. Through a special firmware modification [56], the Arduino can be modified to behave like a regular X-Box gamepad. Thus, native support in any game is ensured.

## 5. Implementation

As the implementation follows the UCD cycle, this section describes the implementation of the third stage. Before explaining the implementation during this iteration, the pros and cons of using GTA V are discussed briefly. Note that all detailed technical information, images, readme files, and the source code can be found in the file archive at https://www.hs-osnabrueck.de/prof-dr-julius-schoening/sim2ride (accessed on 7 February 2023).

The simulator that was created to test the prototypes of the two CET to enhance the safety of a cyclist in traffic uses the video game GTA V to simulate the traffic area. Using GTA V has several advantages, such as the following:

1. GTA V already has an immersive traffic system with various road users and vehicles. Thus, no implementation work of a realistic traffic simulation is necessary.
2. GTA V also provides the environment with realistic 3D assets, textures, animations, and weather effects, which makes the simulator realistic.
3. GTA V comes with a built-in physics system that handles collisions of vehicles realistically enough not to require additional implementation work, again lowering the cost and creation time of the simulator.
4. GTA V has an active modding community, which has identified different ways to change the game’s logic, which means that there might already be a mod that can help run an experiment or serve as inspiration for the experiment code, simplifying the implementation work.
5. GTA V provides various input devices to be connected to the game. They all act as a controller with the original controls, allowing for more realistic input devices, such as modified bicycle handlebars.
6. GTA V is a popular game, and while the city it takes place in is fictional, it is primarily inspired by American cities, invoking a sense of familiarity in players.

Of course, it should be noted that the presented approach has its limitations, such as the following:

1. GTA V does not consider the legal road rules, which differ in many countries.
2. GTA V has, relative to real life, limited interactions between traffic vehicles, pedestrians, and the environment.
3. GTA V does not provide fine-grained operation of the bike. In contrast to the car simulation, the bike simulation is very simplified.
4. GTA V’s storylines can be disturbing because some parts cannot be skipped by modding the game.

Considering the advantages and disadvantages, using GTA V as a base enables a low-cost simulator with high visual and auditory realism. This high degree of realism is especially important for the use-case of traffic safety simulation.

### 5.1. Simulator

**Figure 4:**
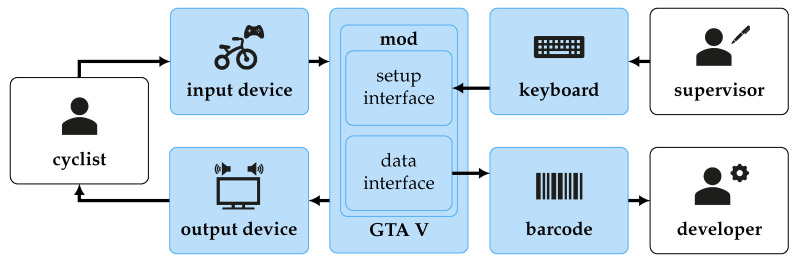
The simulator’s architecture; in blue, the contribution of this work where parts combined and implemented to match the requirements.

A short feasibility study revealed that GTA V is capable of simulating bicycle riding. A program called *Script Hook V* allows one to extend the game with custom scripts. Through the library, a script can access over 5000 native in-game functions [57]. Such a script is developed to implement the simulator. The entire architecture can be seen in Figure 4. The mod script runs inside the GTA V game engine. It offers an interface to quickly set up different traffic situations using function keys on a keyboard.

**Figure A2:**
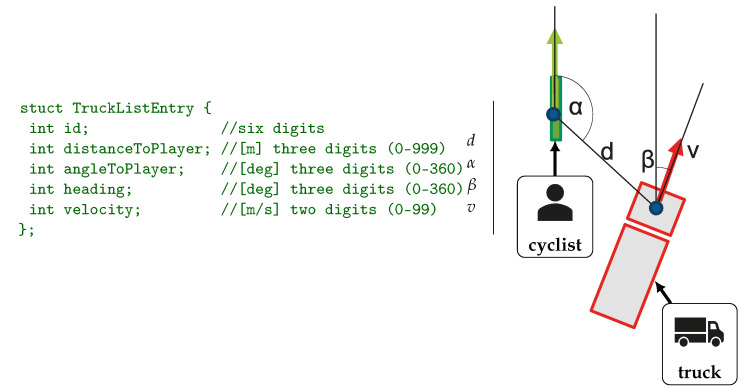
Truck parameters calculated by the mod script.

**Figure A3:**
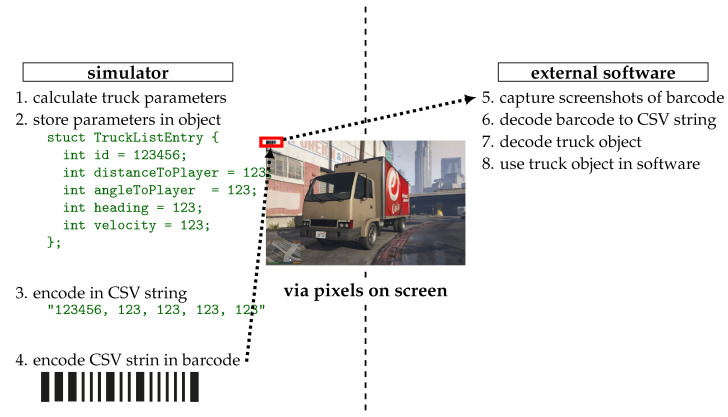
Data transmission from the simulator to external software components through barcodes on the screen.

For the data interface, the mod detects all trucks in a certain radius around the player and captures an agreed-upon set of parameters; see Figure A2. The result is a list of trucks that has to be relayed to the developers. Providing the list of trucks to the developers turned out to be difficult, as GTA V scripts are not allowed to access the file system or any kind of communication utility of the underlying windows operating system. The only way the mod can communicate is through the game utilities. These utilities, for example, allow drawing objects on the screen. Consequently, a routine is developed that translates each entry in the truck list into a barcode that is then drawn to a corner of the screen. The whole process is explained in Figure A3. This solution faces another limitation of the game. A script can draw a maximum of 300 bars per frame to the screen. A serialized truck translates to 168 bars, meaning the list must be transmitted over multiple frames. Transmitted the truck list over several frames leads to a delay in truck list transmission, e.g., if the frame rate is 24 fps and there are 10 trucks in the list, it takes just below half a second to transmit the complete list. This fact does not meet the real-time requirement of providing the truck list. The implementation also requires the developers’ external code to be fast, as any missed frame translates to a missed truck.

### 5.2. Input Device

**Figure 5:**
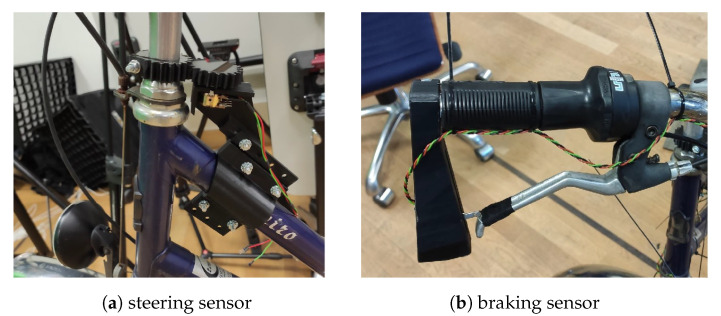
Sensors mounted to (**a**) the head tube and (**b**) the brake lever.

**Figure A4:**
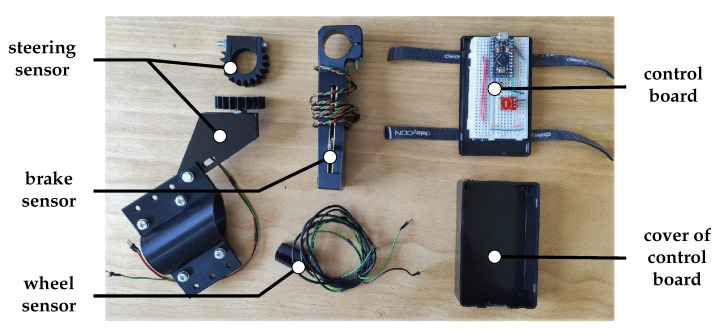
Overview of the controller components.

According to the design concept, a bicycle on a stand is turned into a gamepad controller for a PC running GTA V. The device consists of three sensors, a microcontroller, and various custom-designed 3D printed mounts. Figure 5 shows the mounted steering and braking sensors. An overview of all custom hardware components of the input device is given in Figure A4, and the circuit diagram can be seen in Figure A6. The 3D files for the printed components can also be found in the aforementioned file archive.

**Figure A5:**
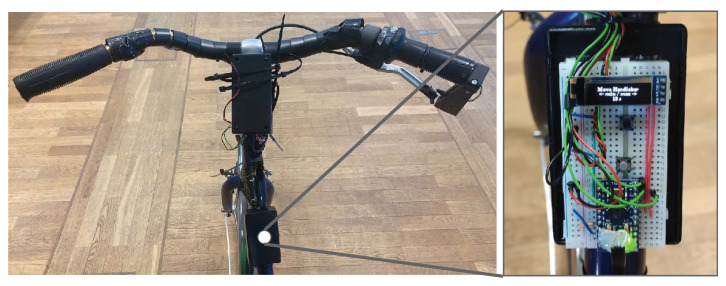
Cyclist’s point of view with the installed sensors and the hidden control box.

**Figure A6:**
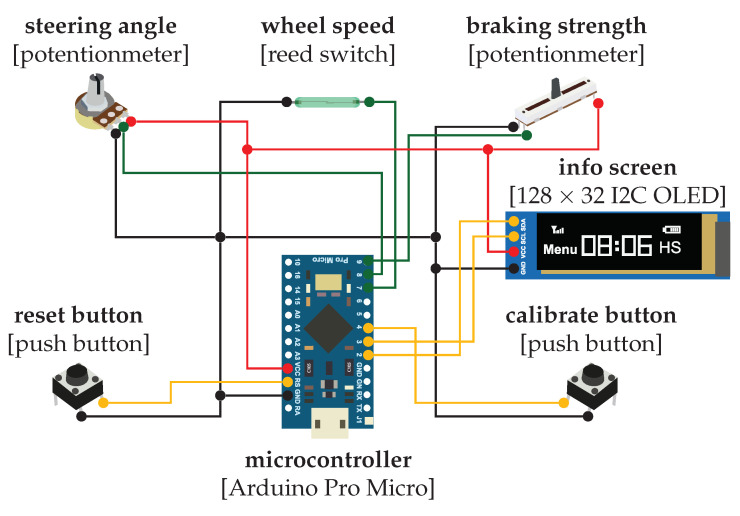
Circuit diagram of the input device—the gamepad controller.

The Arduino is also equipped with a small screen and a button for setting the simulator up. This button and the display allow the supervisor to calibrate the steering sensor. This specialized supervisor interface is hidden by a cover so that it does not disturb the cyclist during an active simulation. All the hardware attached is designed to be discrete and mostly hidden from the cyclist’s point of view, as seen in Figure A5. The Arduino can capture every input with more than 200 discrete steps precision. However, another limitation of GTA V is that the game reduces the input to bicycle steering from −100% to +100% to just five steps. This simplification makes precise steering with an actual handlebar impossible. Thus, a special routine in the firmware is developed to mitigate this problem. The program pulses the input signals to once again turn 5 steps into a range of 200 steps. This solution can sometimes cause camera shakes in the game as a side effect. The speed signal has a similar issue, as the game uses two buttons for normal and extreme speed. However, speed is not the simulation’s focus, so the input is reduced to three steps. The braking input does not require any modification. It is important to note that GTA V features continuous input translation for any vehicle other than the bicycle. The game’s input configuration can also not change bicycle inputs.

### 5.3. Full Setup

**Figure 6:**
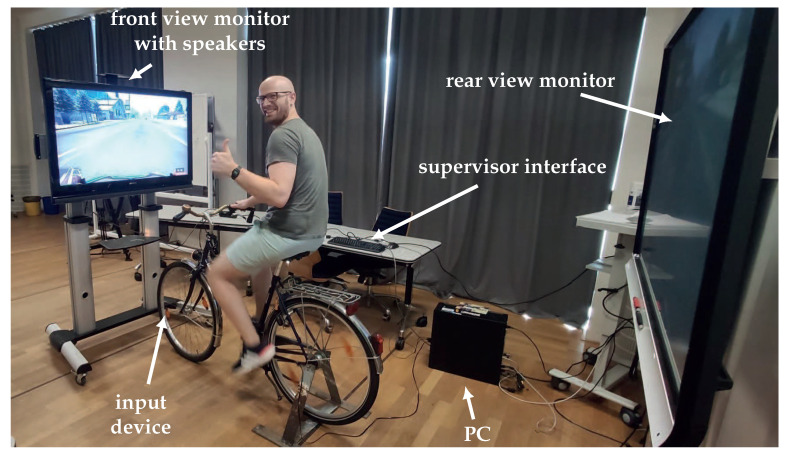
Overview of the full simulator setup.

The complete simulator can be seen in Figure 6. It consists of the modified stationary bicycle as the gamepad connected to a PC running GTA V. The table in the back with a keyboard on it allows the supervisor to control the simulation. The primary monitor in front of the bicycle displays the front view for the cyclists and also outputs the game’s sounds. The secondary monitor in the back is supposed to be a rear-view display. However, GTA V can only render a single camera. For this reason, both screens show the same view, and the supervisor has to manually trigger the rear view through the keyboard whenever the cyclist turns his head around.

## 6. Evaluation

This section describes step four of the UCD cycle, the evaluation. Through a user survey, the current state of the developed simulator is compared to the user requirements. Additionally, the UCD steps one and two are repeated by defining requirements for the next version of the simulator.

### 6.1. User Survey

The simulator is evaluated in combination with the user survey for the CET concept. The cyclist is placed on the simulator and gets five minutes to get used to the riding experience. This warm-up is followed by a few more minutes with the CET concepts. After riding the simulator, the subject is given a questionnaire for the CET concept tested and a second questionnaire for evaluating the simulator. The simulator questionnaire can be seen in the aforementioned file archive. In total, six subjects were questioned.

Feedback was collected based on German questionnaires. The questionnaire consists of a couple of general questions about the subjects. All subjects were between 23 and 37 with a median age of 26.8. The questionnaire then continues with several statements about the simulator that the subject can agree or disagree with. The accumulated result can be seen in Table 3. According to this table, understanding and using the system was easy for the majority of the users. However, some of them had issues with the system, which also reduced their perceived realism. Further comments on the questionnaire gave different explanations for this. These are summarized in the following list:

- Missing input precision.
  - The maximum cycle speed should be faster [2 subjects].
  - More granularity in the speed input [2 subjects].
  - Steering not sensitive enough [1 subject].
- Lack of ability to shift weight in curves leading to dizziness [1 subject].
- Disposition due to low resolution, possibly due to low FPS [1 subject].
- Concerns about the structural integrity of the bicycle stand [1 subject].
- Wish for different cycling modes, e.g., e-bikes and e-scooters [1 subject].

Additionally, subjects were asked to evaluate the simulator based on 13 opposing adjectives. The results can be seen in Table A2. These results reinforce the impression that most subjects found the simulator easy to understand and use. The majority also found the system to be interesting and innovative. It is also clear from the questionnaire that some users found the simulator to be more of a problem than support and more complicated than simple. This result is possible because the realism of the inputs is not satisfactory yet.

### 6.2. Requirements Check

The user requirements defined in Section 3.4 are checked against the system’s current state as seen in Table 1. The user survey shows that the simulator feels safe and easy to learn and use for most users. The input realism turned out to be an issue. Therefore, the input realism is added to the list of requirements and is not yet fully satisfied. The simulation is currently highly repeatable and modifiable for supervisors through a few keyboard strokes. It is also discrete and does not interfere with the user interfaces of other projects, as accentuated by Figure A5. An issue for external developers is the data interface, which is currently strongly coupled to the game frame rate and limited to transmitting information about a single truck per frame. This limited throughput leads to delays and makes it too slow to be a real-time interface.

## 7. Cognitive Enhancement Technologies Concepts for Safe Rides

**Figure 7:**
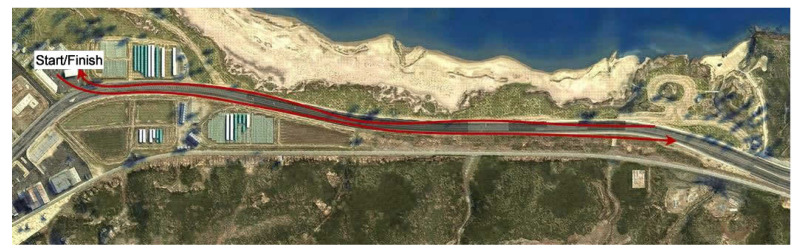
Test track selected for the first evaluation of both CET concepts for safe rides.

Two early-stage CET concepts were piloted and tested using the simulator to allow cyclists to sense dangerous traffic situations. For the evaluation, a specific test track was established. This track seen in Figure 7 was chosen because a particularly large number of trucks appear on this section of the road. It was also decided to use a route that is as straight as possible for the first tests to reduce the more challenging condition of driving around curves. On this stretch of road, the subject was meant to drive on the right side. Motor vehicles, therefore, always pass on the left-hand side. In the case of the tactile dictionary, this resulted in a one-sided use of the vibration signals on the left handle. The block-shaped visualization also favored the left-hand side. All test subjects were aware of this in advance to avoid confusion. The test drive was terminated as soon as ten trucks passed the test person.

### 7.1. Tactile Dictionary on the Handlebar

The subject receives feedback from the bicycle handlebar for the tactile dictionary. Therefore, four vibration motors and a beeper for generating sounds are installed on the handlebar. Vibration motors serve as a warning and the beeper as an alarm. Each end of the handlebar has two vibration motors to encode the general direction of the approaching vehicle and the distance from the cyclist. If a truck comes from the left side, only the left handlebar grip vibrates, and vice versa. A slight vibration begins when the truck is 80 m away and increases at 40 m. In addition to encoding the distance, these vibrations inform the user of an approaching truck as gently as possible. At a distance of 20 m, the beeper generates a sound to alarm the cyclist of a dangerous situation. At this point, the subject should already be aware of the approaching truck due to the vibration and thus not be startled by the alarm signal.

### 7.2. Visual Block-Shaped Dictionary

**Figure 8:**
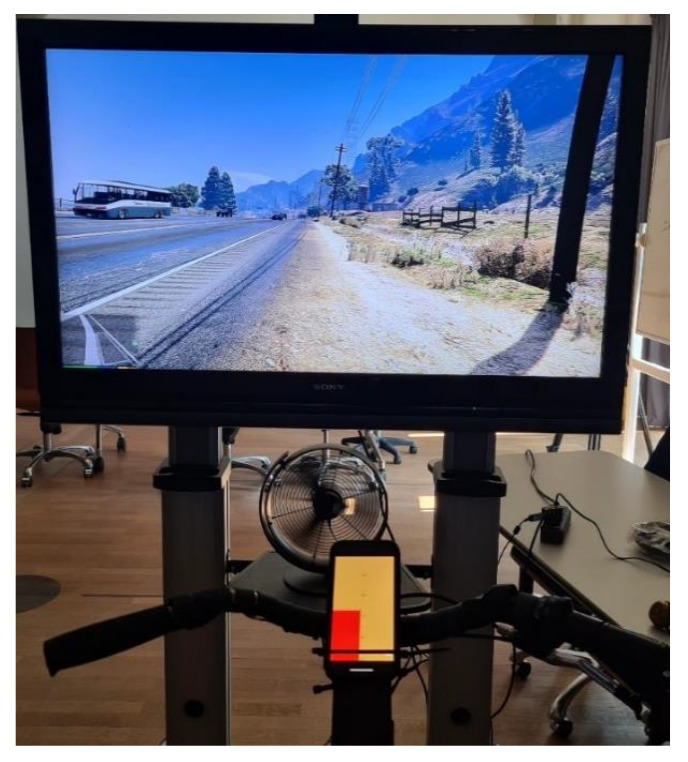
Block-shaped visualization of a truck from left behind from the subject view.

Figure 8 shows the block-shaped visualization in the simulator setup. The subject sees the traffic simulation on the screen in the direction of travel. With the goal of a realistic simulation, a fan is located underneath the screen to simulate airflow while driving. The CET device is a smartphone connected to the bicycle. On the CET device, information about the distance of the nearest truck is encoded in colored columns. The height of the columns corresponds to the distance between the vehicle and the cyclist. A truck on the left of the cyclist fills the left column, and a truck on the right fills the right column. Trucks approaching from the front are ignored, as the user’s field of vision covers this area. Parked trucks are also ignored.

The block-shaped dictionary starts to display at a distance of 80 m from the truck and increases linearly to 0 m. The value of 80 m was determined to be appropriate for the test track and with the maximum speed of the bicycle during the initial self-tests. For a different test scenario, this value can be adjusted with little effort. The speed of bicycles and motor vehicles greatly influences the appropriate detection distance. An alternative form of information for the visual dictionary could have been to display the arrival time of the next truck. However, since estimating the truck’s speed and the time until impact is a critical part of the users’ decision of when to react, the distance must remain the only information. Otherwise, a reliable estimate of the speed by the user would no longer be possible.

### 7.3. Preliminary Results

The feedback from the four subjects while piloting the two early-stage CET concepts to enable cyclists to sense dangerous traffic situations is quite promising. The subjects preferred the tactile dictionary since they did not need to look down at the handlebars to obtain the information. Instead, they could feel vehicles approaching from behind while keeping their other senses focused on the driving task. However, a subject stated that vibrations might be hard to recognize on a ride over cobblestones. In the subjects’ opinion, the block-shaped dictionary was prominent in the field of vision. The subjects tended to be contentious, looking at the display and not focusing on the scene.

During the testing of the CET concepts, balance problems were noticed that could arise due to the fixed-installed bicycle of the simulator. Because only the rear tire rotates, the gyroscopic forces are significantly reduced. Despite the bicycle’s stable stand, it reacts differently to pedaling movements in the experimental setup than a moving bicycle would on the road. Furthermore, the stationary front wheel makes steering movements very smooth and takes some getting used to. In addition, it is impossible to lean into curves with the experimental setup. The aspects mentioned above and the additional noticeable delay between real and virtual steering movements make it challenging to do a curve in some situations.

## 8. Conclusions

The development of advanced computer simulation for traffic areas is very time consuming. While GTA V is a great tool to simulate complex traffic situations in a way that feels realistic to the user, developing mod scripts for the game is particularly complicated due to a lack of documentation. Furthermore, the scripting environment and the game’s limitations on inputs and outputs require a lot of extra integration work. Nonetheless, the developed system allows for a safe simulation of dangerous traffic situations. It is suitable for the context of evaluating CET, as shown by the two CET mentioned, and is easy to set up within two days using the provided aforementioned file archive.

### 8.1. Further Works

The evaluation highlighted that the simulator has multiple weak points and does not fully satisfy all user requirements. To fix the current issues, satisfy all of the user requirements, and create an even more realistic simulation, another full iteration of the UCD cycle needs to be completed. Section 6.2 sets the foundation for that by expanding the user requirements list. As input realism is currently the main reason for cyclist dissatisfaction, improving it should be the main focus. This can be done through more elaborate algorithm tuning in the input device firmware. An alternative way would be to overwrite the game’s default input translation from inside the mod script. Script Hook V offers native functions to do that. Another alternative would be to replace the input device with a smart bike trainer and integrate this with the game through the “GT Bike V” mod [48]. The real-time data interface also requires reworking. Expanding the transmission rate by using multicolored bar codes might be an option. This would allow the use of 16,581,375 different RGB values per bar and, therefore, massively increase the throughput per frame.

### 8.2. Limitations

To create an entirely realistic road simulator, an important factor is the legal road rules, which GTA V as a system does not fulfill. Such traffic laws differ worldwide, meaning that the localization of such a simulator would also be integral. However, in the context of CET, the legality of traffic rules might not be such an important factor as long as the subject experiences the traffic as realistic enough to be immersive. It is possible to create test scenarios, as shown with the exemplary CET, that only need basic traffic behavior, such as stopping at red lights, to allow for an evaluation of this technology. This is where the simulator shines in its current form. Still, because of this traffic-law limit, the GTA V-based simulator is unsuitable as a testing system for all kinds of traffic-oriented technology.

### 8.3. Impact on the Society

As we mentioned in the Introduction, accidents involving cyclists are still a big problem worldwide. Developing bicycle infrastructure, especially in many EU countries, undoubtedly reduces the number of road accidents involving cyclists. However, as the cycling infrastructure develops, it is crucial also to improve the quality of technology for cyclists. Continuous research on optimal parameters for bicycle helmets and equipping them with solutions, such as airbags [58], is one option, but legislative changes to grant cyclists more rights also contribute to greater cyclist safety. The automotive industry already places great emphasis on the use of IT applications and numerous sensors to ensure safety, e.g., parking sensors, distance sensors about the vehicle ahead, and overtaking information systems. However, the use of analogous systems in the bicycle industry is still a novelty. Therefore, we express the conviction that the development of technology, which both affects and enhances our cognitive abilities, will also ensure greater safety on the road. Nevertheless, it should be noted that the impact of these technologies on attention is unknown, and it can be both stimulating and overly involved. Therefore, it is important to carry out numerous tests under controlled and safe conditions, using simulators at various stages of their development.

## Figures and Tables

**Table 1:** List of requirements for the simulator; check boxes indicate the current state—orange check boxes indicate that the users give feedback for improvements that are not implemented yet.

Cyclist		Supervisor/Developer	
● Safe	🗹	● Repeatable	🗹
● Intuitive	🗹	● Modifiable	🗹
●Realism		● Discrete	🗹
● Visual Realism	🗹	● Real-Time Data Interface	🗹
● Auditory Realism	🗹		
● Input Realism	🗹		

**Table 2:** Decision matrix of the simulator, documenting significant design decisions.

	Prop Simulation	Video Playback	Computer Simulation
Visual Realism	−	+	+
Auditory Realism	−	+	+
Interactivity	+	−	+
Simplicity	+	+	−
Repeatability	−	+	+

**Table 3:** Result of the user survey of the simulator; the background color indicates the number of answers, from white—no answer -to dark blue—five answers.

	Totally Agree	Agree	Neither	Disagree	Totally Disagree
The system was easy to use.	5		1		
The system was easy to understand.	5		1		
I would like to use the system every day.	1	1	2	2	
I had issues with the system.			2	3	
The system was too complex.			1	3	2
I quickly learned how to use the system.	3	3			
I would be able to easily learn how to use the system with only a written manual.	3	1	1	1	
I felt safe using the system.	2	1	2		
The system was inconcistent.			2	3	1
Dangerous Situations were simulated realisticly.	1	3	2		
The sytems functions were well integrated.	3	2	1		

## Data Availability

Source code and additional information are contained within the article or can be found in the file archive at https://www.hs-osnabrueck.de/prof-dr-julius-schoening/sim2ride (accessed on 7 February 2023).

## Appendix A

**Table A1:** Input capturing capabilities of existing solutions for bicycle-based input devices.

Input	Example 1 [54]	Example 2 [55]	Example 3 [53]
Speed	yes	yes	yes
Braking	no	no	yes (0 to 100%)
Steering	yes (left,right)	yes (left,right)	yes (−100 to +100%)

**Table A2:** Result of simulator rating by opposite adjectives as a seven-point scale from one opposite adjective to another.; the background color indicates the number of answers, from white—no answer -to dark blue—five answers.

incomprehensible				1	1	4	understandable
creative	2	3		1			uncreative
easy to learn	4	1		1			hard to learn
boring			1	1	3	1	exiting
uninteresting			1		1	3	interesting
original	2	3		1			conventional
hindering		1	1	3		1	supporting
good	3	2	1				bad
complicated		2			2	2	simple
inefficient			2	1		3	efficient
conservative			1		3	2	innovative
clear	3	2	1	1			confusing
clean	3	1	1	1			overloaded
